# Editorialets

**Published:** 1910-03

**Authors:** 


### Editorialets.

DOCTOR, don't pigeonhole that little bill I sent you. Please remit. Do it now.

---

REQUIREMENTS ADVANCED.—The Memphis Hospital Medical College has adopted the standard of the Association of American Medical Colleges.

---

THE "ANNUAL PROGRESS" number of *Clinical Medicine* (January) is, indeed, most creditable to its publishers, and contains a wealth of matter of importance and interest to every physician.

---

THE Missouri Valley Medical Society will meet at Omaha, March 17th and 18th, inst., Dr. A. B. Somers, President. The tumultuous, tremendous, whopping and altogether irresistible Charles Woods Fassett is Secretary. Go.

---

DR. BUTLER'S BOOK, "Treasures of Truth," is, indeed, a gem of purest (optimism) serene. It is a wholesome tonic and simulant and should be taken daily, but in "broken doses"—not to be well shaken before taking; you'll do the shaking. It will make you "smile awhile, and while you smile, another will smile," etc.

S. DeWitt Clough, Publisher, Ravenswood, Chicago. Cloth, 75 cents; leather bound, $1.00.

---

THE alumni of the Medical Department of Tulane University have organized, and at Dallas during the session of the State Medical Association a meeting will be held to perfect the organization. The Secretary-Treasurer, Dr. J. A. Hill, of Houston, has issued a call to all the Tulane fellows to attend. Dr. Russell Caffery, of San Antonio, is President. Let there be a full attendance. Dr. Dyer and Dr. Dock, Tulane professors, will address the meeting.

---

DEATH OF DR. JOSEPH K. COMBE.—This prominent and popular young physician died of pneumonia at his home in Brownsville, January 21st. His death, which was almost sudden, was a great shock to the community and is universally deplored by a host of professional brothers who knew and loved him. Dr. Combe was the son of the late Dr. C. B. Combe, whose death we were called to mourn so recently, and a brother of Dr. Fred J. Combe, mayor of Brownsville. He was born at Matamoras, Mexico, October 16, 1871. He graduated from Notre Dame College, Indiana, in 1893, and from the Medical Department of the University of Virginia in 1896. Soon after he received his degree he was called to New York to accept the position of Assistant House Surgeon of Infant Asylum. From 1898 to 1902 he was Acting Assistant Surgeon U. S. A., in charge of the hospital at Fort Brown and filled many other positions of honor and trust. We shall miss him.

Peace to his ashes.

---

FRIENDS OF THE MEDICAL PROFESSION.—Captain J. C. Ralston, of Waller county, Representative in the last Texas Legislature, was in Austin last week and favored us with a call. Every doctor in Texas has reason to love and respect this noble old Roman, who stood so firmly by the cause of medical legislation, and who, by his personal efforts, secured the passage of all the bills for sanitary reform and advancement that were passed. It is no fault of his that Governor Campbell vetoed the tuberculosis bill, and crippled the State Board of Health bill after it was passed. Captain Ralston was in Austin to work for that grand old statesman—R. V. Davidson—for Governor, the friend of the medical profession and champion of sanitary and medical legislation. I wish to impress upon my readers that there are two Davidsons, a big one and a little one. R. V. is the big one. The little one is named A. B., and is the man, then Senator, who said "Let the doctors dissect each other; damn them and their ghost bill."

---

THE MEXICAN INTERNATIONAL MEDICAL ASSOCIATION held its annual meeting at Aquascalientes, Mexico, February 1 to 3, ult. Dr. Howard Kelley, of Baltimore, was present and read a paper. Dr. R. H. L. Bibb, the Nestor of medicine in Mexico, was elected President for 1911. This honor was accorded Dr. Bibb, says the Secretary, on account of his earnest and enthusiastic work for the Association and his high ethical standing in the medical profession. The next meeting will be held, nominally, in Juarez, Chihuahua, but really in El Paso, Texas. The officers elected are Dr. C. E. Husk, Santa Barbara, Chihuahua, President; Dr. F. W. Taube, Zacatecas, Vice-President; Dr. J. S. Steele, Monterey, Secretary and Treasurer (re-elected); Dr. H. D. Eaton, Chihuahua, and Dr. R. Grieve, Santa Barbara, Censors. Dr. R. D. Robinson and Dr. W. R. Jameson, of Torreon, editors of the "Annual." Honorary members, Drs. W. R. Bramer, El Paso; B. L. Wright, Las Animas; Col. and J. A. Lamb, of Kalispell, Mont. Socially the doctors had a fine time, as well as a most enjoyable and profitable meeting. "Jack" Steele is a Texas boy and is an electric motor flying machine and a Buick auto combined, forty horsepower.

